# Is aripiprazole a key to unlock anorexia nervosa?: A case series

**DOI:** 10.1002/ccr3.3271

**Published:** 2020-09-06

**Authors:** Akın Tahıllıoğlu, Tuğçe Özcan, Gamze Yüksel, Noorjahan Majroh, Sezen Köse, Burcu Özbaran

**Affiliations:** ^1^ Department of Child and Adolescent Psychiatry Faculty of Medicine Ege University Izmir Turkey

**Keywords:** anorexia nervosa, aripiprazole, BMI, case series, depression, follow‐up, treatment

## Abstract

Aripiprazole contributes an increase in body mass index and attenuation in anorexia nervosa (AN) symptoms, leading clinical improvements with lower side‐effect profile; but it is not enough to cure comorbid depressive symptoms in AN.

## Graphical abstract

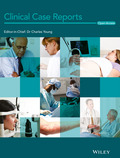

## 1 INTRODUCTION

        

Anorexia nervosa (AN) is an eating disorder in which the person has a significantly lower body weight in view of age, sex, development, and body health due to limitation of energy intake. In AN, the desire to have a thin body despite the low body weight and the excessive fear of gaining weight push the patient to various specific behaviors to lose weight. The main problem is about perceiving body weight or shape. When assessing oneself, the person attaches undue importance to body weight and shape or cannot comprehend the importance of low body weight at that time.^1^ AN is also associated with the risk of developing lifetime psychiatric comorbidity.^2^

Anorexia nervosa may be comorbid with mood disorders, especially major depression, anxiety disorders, obsessive‐compulsive disorder, autism spectrum disorder, attention‐deficit/ hyperactivity disorder (ADHD), personality disorders, and substance use disorder.^3^ Psychotic symptoms may appear temporarily during the course of AN, but studies showing comorbid psychotic disorders are also present.^4^, ^5^, ^6^

The clinician's priority in the treatment is to ensure adequate caloric intake and correction of malnutrition.^7^ Both individual and family‐oriented psychotherapies constitute an important part of the treatment. Although there is currently no approved medication by the FDA (The Food and Drug Administration) in the treatment, medication (especially olanzapine) may be necessary for patients with excessive anxiety and agitation.^8^ In a review conducted in 2016, studies using typical/atypical antipsychotics, selective serotonin reuptake inhibitors (SSRIs), tricyclic antidepressants (TAD), monoamine oxidase inhibitors (MAOI), mood stabilizers, benzodiazepines, alpha 2 adrenergic agonists, and many other agents were examined. However, strong evidence of treatment efficacy for AN could not be obtained.^9^

Aripiprazole is an atypical antipsychotic medicine that has partial agonistic effects on D2, D3, and 5HT1A receptors and antagonistic effects on 5HT2 receptors. It has FDA approval for use in the treatments of manic or mixed episodes associated with bipolar disorder, Tourette's syndrome, autism‐related irritability, schizophrenia, and resistant depressive disorders.^10^ Researches on neurobiology suggest that brain reward circuits are hypersensitive in AN, which may be associated with hypersensitivity in the dopamine system and may provide treatment goals.^11^, ^12^, ^13^ Aripiprazole is thought to lead to improvements in brain functions by regulating D2 receptor activity.^14^ In a study conducted in patients with AN, weight gain, decrease in obsessions associated with body image and food, improvement in comorbid depression or anxiety, and increase in cognitive flexibility were shown with the use of aripiprazole.^15^ There are case series showing that aripiprazole treatment in AN is associated with an increase in body mass index.^16^

When the literature is reviewed, there are a limited number of studies, case reports, and case series about aripiprazole in the treatment of AN^15^, ^17^ (See Table 1). Although it is a commonly shared opinion that aripiprazole treatment reduces obsessive eating attitudes and comorbid depressive symptoms in AN, it is difficult to find evidence‐based data related to these improvements. In addition, the low side‐effect profile of aripiprazole is thought to be beneficial in improving the treatment compliance for young people with AN who usually have treatment compliance problems. In light of the findings and clinical observations in the literature, this study aimed to evaluate the changes in body mass indexes, depression severity, clinical improvement scores, and drug‐related side effects before and after aripiprazole treatment in patients with AN who were treated with aripiprazole.

**TABLE 1:** Studies investigating the effectiveness of aripiprazole in AN

Author(s)	Publication year	Age	Number of patients	Dosage of aripiprazole	Duration	Result
Trunko et al	2011	15‐55	8 (5 with AN, 3 with BN)	5‐15 mg/d	4‐41 mo	Decrease in fear of eating, obsessional thoughts, increase in BMI
Frank	2016	12‐17	4	2‐5 mg/d	2‐4 wk	Improve in body image, decrease in fear of eating
Frank et al	2017	14.4^a^ (ARI −) 15.0^a^ (ARI +)	84 (ARI −) 22 (ARI +)	1‐5 mg/d	15.9^a^ d (ARI −) 18.9^a^ d (ARI +)	Increase in BMI, weight gain

Abbreviations: AN, anorexia nervosa; ARI, aripiprazole; BMI, body mass index; BN, bulimia nervosa.

^a^ Mean value.

## 2 METHODS

The study was designed as a retrospective case study. Eleven girls aged 11‐17 years who applied to the Ege University Department of Child and Adolescent Psychiatry between 2017 and 2019 and continued their clinical follow‐up for at least 20 months with anorexia nervosa were included in the study. Ethical committee approval for the study was received from the Medical Research Ethics Committee of the Ege University (Decision no: 19‐8.1T/54). The cases and their parents were informed about the use of their medical information in the study, and written consent was obtained from them.

### 2.1 Case selection

Patients aged 11‐17 years who fulfilled the psychiatric diagnosis of anorexia nervosa according to Diagnostic and Statistical Manual of Mental Disorders‐5 (DSM‐5) criteria and regularly used “aripiprazole” were included. Patients with autism, mental retardation, psychotic disorder, bipolar disorder, and/or substance use disorder were excluded from the study.

First psychiatric examinations of the patients were carried out by assistant doctors (A.T, T.Ö, G.Y, N.M) who had sufficient clinical experience. To clarify the current diagnoses and comorbidities, a second psychiatric interview was conducted by two academic doctors (B.Ö, S.K) who had at least 15 years of clinical experience.

### 2.2 Evaluation materials and case follow‐up process

After completion of the case selection procedure, clinical diagnoses of the patients, comorbidities, sociodemographic information, all medical treatments chronologically applied to each case, initial and highest doses, side effects and benefits, transition to aripiprazole, initial and highest doses of aripiprazole, benefits, duration of use and the presence of side effects were noted. Eating disorder symptoms were determined by psychiatric examinations based on DSM‐5 criteria. Clinical Global Impression Scale (CGI),^18^ Beck Depression Inventory (BDI),^19^, ^20^ and body mass index (BMI) were used for the evaluation of the cases. In each case, a multidisciplinary approach was carried out within the scope of psychopharmacological treatment arrangements, interviews with nutritionists, supportive psychotherapies, and pediatric consultations when needed. Body mass index (BMI) scores of the patients were measured at the beginning and the end of the treatment.

### 2.3 Statistical analysis

The SPSS (The Statistical Package for Social Sciences) 22.0 program was used to analyze whether the data obtained from the CGI, BDI scores, and the mean BMIs of the cases differed significantly before and after treatment. Kolmogorov‐Smirnov test was used to determine whether the quantitative variables were suitable for normal distribution. In terms of variables with normal distribution, the groups were compared with two independent samples t test. The groups were compared with the Mann‐Whitney U test for variables not showing normal distribution. *P* < .05 was considered statistically significant.

## 3 RESULTS

The ages of the 11 patients ranged from 11 years 8 months to 17 years at the time of the first admission, with a mean age of 14.3 years. The follow‐up period of the patients in our clinic lasted for 20‐28 months. The mean follow‐up period was 23.7 months. All the patients were followed up in our outpatient clinic, and none of them had a history of inpatient treatment in a psychiatric service. Descriptive features of the cases such as familial characteristics and the comorbidities of the patients are shown in Table 2.

**TABLE 2:** Familial characteristics and comorbidities of the cases with AN

Cases with anorexia nervosa (n = 11)	n (%)
Presence of familial psychopathology	
Yes	6 (54.6)
No	5 (45.4)
Education level of mother (year)	
0‐5 y	2 (18.1)
6‐8 y	0 (0)
9‐11 y	4 (36.3)
>12 y	5 (45.4)
Education level of father (year)	
0‐5 y	1 (9)
6‐8 y	0 (0)
9‐11 y	2 (18.1)
>12 y	8 (72.7)
Comorbid psychiatric diagnoses of the cases	
Major depression	10 (90.9)
Attention‐deficit/Hyperactivity disorder	4 (36.3)
Anxiety disorders	2 (18.1)
Obsessive‐compulsive disorder	2 (18.1)
Social phobia	1 (9)

### 3.1 Clinical follow‐up findings

Pharmacological treatment history and data related to the usage of aripiprazole are shown in Table 3. Nine of the 11 cases had antipsychotic medication at the beginning of the treatment, four of them had risperidone (n = 4; 44.4%), three of them had olanzapine (n = 3; 33.3%), and the remaining two had aripiprazole (n = 2; 22.2%).

**TABLE 3:** Treatment history and use of aripiprazole in the cases with AN

	Comorbid psychiatric diagnoses	First medication treatment	First medication treatment maximum doses	Side effects with first medication treatment	Reason for stopping the first medication treatment	Initial dose of aripiprazole	Maximum dose of aripiprazole	Duration of aripiprazole use	Side effects due to aripiprazole
Case 1	MDD, ADHD	Sertraline, Risperidone	Sertraline 150 mg/d, Risperidone 2 mg/d	None	Continued with first medication	5 mg/d	5 mg/d	18 mo	None
Case 2	MDD, ADHD	Olanzapine, Fluoxetine^a^, Risperidone	Olanzapine 5 mg/d, Sertraline^a^ 75 mg/d, Risperidone 1 mg/d	Galactorrhea	Galactorrhea (Risperidone)	2 mg/d	10 mg/d	20 mo	Increased appetite
Case 3	MDD, Anxiety Disorder	Sertraline, Olanzapine	Sertraline 125 mg/d, Olanzapine 2.5 mg/d	None	Reduction of symptom severity (Olanzapine)	1.25 mg/d	2.5 mg/d	19 mo	None
Case 4	MDD	Sertraline, Olanzapine	Sertraline 200 mg/d, Olanzapine 7.5 mg/d	Increased appetite	Increased appetite (Olanzapine)	2.5 mg/d	15 mg/d	28 mo	None
Case 5	MDD, ADHD, Anxiety Disorder	Sertraline, Olanzapine	Sertraline 100 mg/d, Olanzapine 5 mg/d	Increased appetite	Increased appetite (Olanzapine)	1.25 mg/d	5 mg/d	16 mo	None
Case 6	MDD	Fluoxetine, Aripiprazole	Fluoxetine 40 mg/d, Aripiprazole 1.25 mg/d	None	Continued with first medication	1.25 mg/d	10 mg/d	20 mo	None
Case 7	MDD	Escitalopram, Risperidone	Escitalopram 20 mg/d, Risperidone 1 mg/d	Sedation, increased appetite	Sedation and increased appetite (Risperidone)	2.5 mg/d	7.5 mg/d	17 mo	Sedation
Case 8	MDD, OCD	Fluoxetine	Fluoxetine 30 mg/d	None	Insufficient benefit	1 mg/d	5 mg/d	24 mo	None
Case 9	None	Fluoxetine, Aripiprazole	Fluoxetine 40 mg/d, Aripiprazole 7.5 mg/d	None	Remission	2.5 mg/d	7.5 mg/d	19 mo	None
Case 10	MDD, ADHD, Social Phobia	Fluoxetine, Risperidone	Fluoxetine 30 mg/d, Risperidone 1 mg/d	None	Insufficient benefit	2.5 mg/d	5 mg/d	27 mo	None
Case 11	MDD	Fluoxetine	Fluoxetine 20 mg/d	None	Continued with first medication	0.5 mg/d	3 mg/d	16 mo	None

Abbreviations: ADHD, attention‐deficit/hyperactivity disorder; MDD, major depressive disorder; OCD, obsessive‐compulsive disorder.

^a^ When the Case‐2 applied to our outpatient clinic for the first time, we learned that she had been using Fluoxetine at least 6 mo without any benefit. Therefore, we switched the antidepressant medication to Sertraline.

In the first stage of pharmacological treatment, four of the 11 cases had medication alterations due to side effects (galactorrhea due to risperidone [n = 1]; sedation and increased appetite due to risperidone [n = 1]; and increased appetite due to olanzapine [n = 2]; Cases—2—4—5 and 7). In two cases, the first treatment (fluoxetine and risperidone) did not show any benefit, so the treatments were changed (Case‐8 and Case‐10). In three cases, despite the benefits of the first treatment, aripiprazole was added to the first treatment to accelerate the clinical improvement (Cases—1—3 and 11). In the remaining two cases, since the initial treatment of fluoxetine + aripiprazole was shown to be beneficial, no treatment modification was performed (Case‐6 and Case‐9).

### 3.2 Aripiprazole usage and clinical outcomes

All the cases included in the study used aripiprazole. Aripiprazole treatment duration ranged from 18 to 28 months. The mean initial dose of aripiprazole was 2.02 mg/d (minimum 0.5 mg/d; maximum 5 mg/d). The highest mean dose of aripiprazole used in the cases was 6.86 mg/d (minimum 2.5 mg/d; maximum 15 mg/d; see Table 3). In 27.2% (n = 3) of the cases, the maximum dose of aripiprazole was 10 mg/d or more, and in the remaining 72.7% (n = 8), the maximum dose was less than 10 mg/d.

No side effects were observed in seven of nine patients (77.7%) in whom aripiprazole was subsequently added to their treatment. One of the remaining two cases (22.2%) had increased appetite due to aripiprazole (Case‐2); the other case had sedation that started in the previous treatment and continued with aripiprazole (Case‐7). In two of the four cases (50%) who had switched to aripiprazole treatment due to side effects of the previous treatment, the side effects disappeared completely (Case‐4 and Case‐5). Among 11 cases using aripiprazole, an aripiprazole‐induced side effect was identified in only one case (n = 1, 9.1%; Case‐2) and this was an increase in appetite.

Fluoxetine was the most commonly used antidepressant in the initial treatment (54.5% of the cases, [n = 6]), whereas after switching to aripiprazole, the most commonly preferred antidepressant used with aripiprazole was sertraline (54.5% of the cases [n = 6]); therefore, it was observed that antidepressant which was frequently preferred in the beginning and the end of the treatment was different. Data on treatment history and the use of aripiprazole are shown in Table 3.

There were clinically significant improvements in obsessive eating attitudes and behaviors in all patients. While the mean BMI (BMI1) at the beginning of treatment was 16.02, the mean BMI (BMI2) increased to 20.63 at the end of follow‐up. In nine (81.8%) of the 11 patients, the difference between the last and the first BMIs was over 3. BMI changes were statistically significant (*P* < .001, *t* = −5.474).

The mean CGI‐ “disease severity” score (CGIS1) of the 11 patients at the beginning of the treatment was 6.09 points (between “severely ill” and “very extremely ill”), whereas the mean score at the end of follow‐up (CGIS2) was 1.82 (“normal, not at all ill”). The change in the CGIS scores was statistically significant (*P* < .001, *t* = 11.017). While the mean “improvement” score was 2 (“much improved”) for 11 cases, the mean CGI‐ “side effect severity” score was 1.36 points (between “none” and “not significantly interfere with patient's functioning”).

In addition, the mean BDI score (BDI1) before the treatment was 21.36, whereas in the last session, the mean BDI score (BDI2) reduced to 16.09 points. However, the difference between BDI1 and BDI2 scores was not statistically significant (*P* = .249, *t* = 1.188). The changes in disease severity during the treatment period, depression scores, BMIs, the degree of improvement of the patients with treatment, the current medications, and the severity of the side effects are demonstrated in Table 4.

**TABLE 4:** Changes in BMIs, depression scores and disease severity after treatment in patients with AN using aripiprazole; side‐effect severity and current drug treatments in clinical follow‐ups

	BMI value^a^, ^*^		CGI “Disease Severity” (CGI‐S) Score^a^, ^*^		BDI score^a^, ^*^		CGI “Improvement”	CGI “Disease Severity”	Current drug treatments
	BMI1	BMI2	CGIS1	CGIS2	BDI1	BDI2			
Case 1	17	17.3	7	4	43	31	3	1	Sertraline 150 mg/d, Risperidone 1.5 mg/d, Aripiprazole 5 mg/d
Case 2	18.7	22.4	5	2	34	27	2	2	Sertraline 50 mg/d, Aripiprazole 10 mg/d
Case 3	16.8	22.5	5	1	24	14	1	1	Remission
Case 4	15.3	20.7	6	2	2	4	3	1	Sertraline 50 mg/d, Aripiprazole 7.5 mg/d
Case 5	16.4	22.3	6	1	14	26	2	1	Sertraline 100 mg/d, Methylphenidate 27 mg/d, Aripiprazole 5 mg/d
Case 6	15	20.6	5	2	19	19	2	2	Remission
Case 7	15.6	20.7	6	3	20	0	2	3	Escitalopram 20 mg/d, Aripiprazole 5 mg/d
Case 8	16.5	20.1	6	1	19	12	1	1	Sertraline 50 mg/d
Case 9	13.5	17.3	7	1	24	14	1	1	Remission
Case 10	13.1	24.1	7	1	13	7	2	1	Aripiprazole 1.25 mg/d, Methylphenidate 40 mg/d, Sertraline 75 mg/d
Case 11	18.4	19	7	2	23	23	3	1	Fluoxetine 20 mg/d, Aripiprazole 3 mg/d
*P* a, *	<.001		< .001		.249				
*t*	−5.474		11.017		1.188				

Abbreviations: BDI, Beck Depression Inventory; BDI1, Beck Depression Inventory score at the beginning of the treatment; BDI2, Beck Depression Inventory score at the end of follow‐up; BMI, body mass index; BMI1, body mass index at the beginning of the treatment; BMI2, body mass index at the end of follow‐up; CGI, Clinical Global Impression Scale; CGIS1, Clinical Global Impression Scale‐Disease severity score at the beginning of the treatment; CGIS2, Clinical Global Impression Scale‐Disease severity score at the end of follow‐up.

^a^ Independent samples *t* test was performed to compare BMI, CGIS‐S, and BDI scores of the patients before and after treatment.

* *P* value <.05 indicates a statistically significance.

## 4 DISCUSSION

In this study, the changes in the course of the disease as a result of aripiprazole use together with other antipsychotics and antidepressants in 11 female adolescents with anorexia nervosa were investigated. The data obtained from the study showed that reductions in the symptoms and clinical improvements were seen in all the patients. It was found that there were more treatment‐related side effects and insufficient reduction in the symptoms of the disease in patients who did not start treatment with aripiprazole at the beginning compared to the patients who started the treatment with aripiprazole. When the treatments were replaced with aripiprazole, there were fewer side effects and more clinical benefits. In addition, an increase in BMIs was observed in all the patients who used aripiprazole. Moreover, the severity of depressive symptoms decreased in most of the patients with AN (63.6%) with aripiprazole.

Studies on the neurochemistry and neurobiological processes of AN show that dopamine and dopamine receptors play an important role on the neurobiological basis of AN. D1 and D2 receptors can lead to illusion in body perception, and D1 and D2 receptor hypersensitivity may be associated with hunger sense and binge eating attack. Moreover, D2 receptors may play a role in cognitive flexibility and dopamine receptor agonists increase the learning process and reduce anxiety in patients with low weight and low estrogen levels.^21^ Parallel to these implications, it is considered that dopamine D2 receptors have effects on energy homeostasis, leptin signal, and body composition, which contributes to the weight gain effect. In this way, it is thought to be involved in metabolic mechanisms that increase weight gain.^22^, ^23^, ^24^ For these reasons, the role of dopamine receptors in AN is undeniably important. The emergence of associations between dopamine‐dopamine receptors‐AN trio revealed that medications acting on dopamine receptors may be beneficial for the symptoms of anorexia nervosa, and few studies supported this hypothesis. Consistent with this hypothesis, clinical improvement in AN patients was observed with aripiprazole in our study.

Unlike other atypical antipsychotics, having partial agonistic effects on dopamine receptors, increasing treatment adherence due to low side‐effect profile, aripiprazole attracted attention and aripiprazole usage in AN has become more visible in the literature in recent years. Aripiprazole is considered to improve brain function by regulating the dopamine system which is thought to be hypersensitive in AN.^13^, ^14^ In addition, it is stated that aripiprazole helps to improve insight and behavioral changes.^16^ Furthermore, aripiprazole treatment has been reported to be associated with an increase in BMI in patients with AN.^16^ In our study, it was observed that obsessive and inappropriate eating behaviors such as calorie calculation, calorie restriction, binge eating decreased, and BMI increased in the clinical follow‐ups in the patients who started treatment with aripiprazole or who switched to aripiprazole treatment after the initial treatment.

In our study, the changes in terms of benefits and side effects after antipsychotic medications were found to be consistent with the literature. In four of the seven patients (Cases—2—4—5 and 7) who have initiated treatment with an antipsychotic (risperidone and olanzapine) other than aripiprazole, side effects that interfered with the compliance were observed and treatments were switched to aripiprazole. Aripiprazole was added to the treatment due to needs arising from clinical findings such as insufficient benefit from the initial treatment or the desire to reduce obsessive eating behaviors in the remaining three cases (Cases—1—3 and 10). When side effects were evaluated in patients after the use of aripiprazole, the rate of adverse effects observed with the use of aripiprazole was quite low (aripiprazole caused elevated appetite in only one case of 11 cases [Case—2]). It can be considered that this situation increases the treatment compliance and increases benefits from medication and also enables patients to cope with fewer metabolic complications due to fewer side effects.

In a double‐blind placebo‐controlled study conducted in adolescents and young adults in order to investigate the use of risperidone in AN, there were no significant differences between placebo and risperidone‐treated groups in terms of improvement in body perception, decrease on anxiety symptoms and shortening on recovery time. Also, some side effects like fatigue and dizziness were observed more frequently in the risperidone group than in the placebo group. Besides, this study indicated there were significant increases in prolactin levels in the risperidone‐treated group.^25^ In a placebo‐controlled study about olanzapine use in adolescents with AN, an increase in fasting glucose and insulin levels was reported in the olanzapine‐treated group.^26^ It was also stated that risperidone, olanzapine, and amisulpride had no significant effect on body mass index and dissatisfaction with body shape in a meta‐analysis of AN.^27^ Briefly, more studies are needed on the efficacy of second‐generation antipsychotics such as risperidone and olanzapine, and the data show that these medications do not have sufficient satisfactory efficacy in AN and make it difficult to adapt to treatment because of possible side effects.^28^, ^29^

In the literature, there is a debate about which agent or combinations have a higher effect size in the psychopharmacological treatment of AN. A study shows that SSRI + aripiprazole combination therapy is more effective in reducing inappropriate eating attitudes and rituals when compared with SSRI monotherapy and SSRI + olanzapine combination therapy.^30^ The data obtained in our study support this study. Two of 11 patients (Case—6 and Case—9) who started treatment with SSRI + aripiprazole combination were in remission without requiring any other medication treatment. Moreover, aripiprazole was added in two patients (Case—8 and Case—11) whose initial treatment included SSRI monotherapy to increase clinical benefits. Besides, treatment was changed to SSRI + aripiprazole in seven patients who started treatment with SSRI + olanzapine/risperidone combination due to insufficient clinical benefit or side effects. During the follow‐up, one of these seven patients had remission (Case—3), and the other six showed significant improvements in AN symptoms.

In 2007, Aripiprazole was approved by the FDA for usage in depressive disorder. However, there is no strong evidence in the literature regarding the extent to which aripiprazole improves the depressive symptoms of patients with AN. It was reported that depressive symptoms regressed after treatment with SSRI and antipsychotics including aripiprazole in a group of patients with AN and bulimia nervosa in a case series of 2011.^16^ In a study investigating the combination of augmentation with aripiprazole in patients with AN, it was suggested that SSRI + aripiprazole combination significantly reduced depressive symptoms.^30^ In our study, although the mean depression score after treatment decreased compared to the mean score before treatment of 11 cases, this reduction was not statistically significant. This situation may be related to the low number of cases or the psychopathological nature of the sample. It also can be explained by the fact that although aripiprazole is found to be beneficial in depression, it might not an appropriate choice for depression in patients with AN.

There are some strengths and limitations of our study. The first strength of the study is the fact that the cases consist of only AN without any other specified eating disorders or bulimia nervosa cases. Moreover, the clinicians in the study closely followed the cases and recorded the changes regarding patients’ psychiatric conditions. Looking at the limitations of the study, the findings cannot be generalized because of the small sample size. Besides, since all patients included in the study had multiple medications, it was difficult to determine and predict which improvements in AN symptoms were the direct effects of aripiprazole. Therefore, double‐blind placebo‐controlled studies are needed to eliminate this limitation.

To conclude, both pharmacological and nonpharmacological (nutritional and psychotherapeutic) interventions have a role in the treatment of anorexia nervosa. Agents used in psychopharmacological treatment provide to improve the psychopathologies accompanying AN. Aripiprazole is becoming a more preferred alternative in treatment in terms of decreasing obsessive eating attitudes and increasing BMI in AN. In this case series, the use of aripiprazole increased the BMIs of the cases and decreased the core clinical findings related to AN. On the other hand, it was observed that depressive symptoms of AN patients receiving aripiprazole did not decrease significantly. Compared with other antipsychotic agents, aripiprazole had fewer side effects in patients with AN and, as a result, improved treatment compliance. These findings should be replicated and studied with larger sample groups in order to determine the efficacy and role of aripiprazole in the treatment of AN more clearly.

## CONFLICT OF INTEREST

No authors declare that they have any potential conflict of interest.

## AUTHOR CONTRIBUTIONS

AT: contributed to the processes of investigation, validation, formal analysis, original draft preparation, and review and editing. TÖ: contributed to the processes of investigation, validation, original draft preparation, and review and editing. GY: contributed to the processes of investigation, validation, and original draft preparation. NM: contributed to the processes of investigation and validation. SK: contributed to the processes of investigation, validation, methodology, and supervision. BÖ: contributed to the processes of conceptualization, methodology, investigation, validation, review and editing, and supervision.

## Data Availability

We have not shared the data from this study yet.

## References

[1] American Psychiatric Association . DSM‐V: Diagnostic and Statistical Manual of Mental Disorders. Washington, DC: American Psychiatric Press; 2013.

[2] Jagielska G , Kacperska I . Outcome, comorbidity and prognosis in anorexia nervosa. Psychiatr Pol. 2017;51(2):205‐218. 28581532 10.12740/PP/64580

[3] Marucci S , Ragione LD , De Iaco G , et al. Anorexia nervosa and comorbid psychopathology. Endocr Metab Immune Disord Drug Targets. 2018;18:316. 29437020 10.2174/1871530318666180213111637

[4] Aragona M . Tolerability and efficacy of aripiprazole in a case of psychotic anorexia nervosa comorbid with epilepsy and chronic renal failure. Eat Weight Disord. 2007;12(3):e54‐e57. 17984630 10.1007/BF03327643

[5] Seeman MV . Eating disorders and psychosis: seven hypotheses. World J Psychiatry. 2014;4:112‐119. 25540726 10.5498/wjp.v4.i4.112 PMC4274583

[6] Morylowska‐Topolska J , Ziemiński R , Molas A , et al. Schizophrenia and anorexia nervosa ‐ reciprocal relationships. A literature review. Psychiatr Pol. 2016;54:1‐10. 10.12740/PP/OnlineFirst/63514 28581536

[7] Moskowitz L , Weiselberg E . Anorexia nervosa/atypical anorexia nervosa. Curr Probl Pediatr Adolesc Health Care. 2017;47(4):70‐84. 28532965 10.1016/j.cppeds.2017.02.003

[8] Herpertz‐Dahlmann B . Treatment of eating disorders in child and adolescent psychiatry. Curr Opin Psychiatry. 2017;30(6):438‐445. 28777106 10.1097/YCO.0000000000000357

[9] Frank GK , Shott ME . The role of psychotropic medications in the management of anorexia nervosa: rationale, evidence and future prospects. CNS Drugs. 2016;30(5):419‐442. 27106297 10.1007/s40263-016-0335-6 PMC4873415

[10] Casey AB , Canal CE . Classics in chemical neuroscience: aripiprazole. ACS Chem Neurosci. 2017;8(6):1135‐1146. 28368577 10.1021/acschemneuro.7b00087 PMC5495458

[11] DeGuzman M , Shott ME , Yang TT , Riederer J , Frank GK . Association of elevated reward prediction error response with weight gain in adolescent anorexia nervosa. Am J Psychiatry. 2017;174(6):557‐565. 28231717 10.1176/appi.ajp.2016.16060671 PMC5607032

[12] Frank GK . The perfect storm ‐ a bio‐psycho‐social risk model for developing and maintaining eating disorders. Front Behav Neurosci. 2016;10:44. 27014006 10.3389/fnbeh.2016.00044 PMC4785136

[13] Frank GK , Reynolds JR , Shott ME , et al. Anorexia nervosa and obesity are associated with opposite brain reward response. Neuropsychopharmacology. 2012;37(9):2031‐2046. 22549118 10.1038/npp.2012.51 PMC3398719

[14] Czoty PW , Gage HD , Garg PK , Garg S , Nader MA . Effects of repeated treatment with the dopamine D2/D3 receptor partial agonist aripiprazole on striatal D2/D3 receptor availability in monkeys. Psychopharmacology. 2014;231(3):613‐619. 10.1007/s00213-013-3274-7 PMC3969775 24077804

[15] Trunko ME , Schwartz TA , Duvvuri V , Kaye WH . Aripiprazole in anorexia nervosa and low weight bulimia nervosa: case reports. Int J Eat Disord. 2011;44(3):269‐275. 20186719 10.1002/eat.20807

[16] Frank GK , Shott ME , Hagman JO , Schiel MA , DeGuzman MC , Rossi B . The partial dopamine D2 receptor agonist aripiprazole is associated with weight gain in adolescent anorexia nervosa. Int J Eat Disord. 2017;50(4):447‐450. 28334444 10.1002/eat.22704 PMC5392387

[17] Frank GK . Aripiprazole, a partial dopamine agonist to improve adolescent anorexia nervosa‐A case series. Int J Eat Disord. 2016;49(5):529‐533. 26593328 10.1002/eat.22485

[18] Guy W . Clinical global impression scale. The ECDEU Assessment Manual for Psychopharmacology‐Revised. Volume DHEW Publ No ADM 76, 338, 218‐222.

[19] Beck AT . An inventory for measuring depression. Arch Gen Psychiatry. 1961;4:561‐571. 13688369 10.1001/archpsyc.1961.01710120031004

[20] Hisli N . Beck Depresyon Envanteri'nin geçerliliği üzerine bir çalışma. Psikoloji Dergisi. 1988;6:118‐122.

[21] Frank GK . Could dopamine agonists aid in drug development for anorexia nervosa? Front Nutr. 2014;1:19. 25988121 10.3389/fnut.2014.00019 PMC4428488

[22] Perez Millan MI , Luque GM , Ramirez MC , et al. Selective disruption of dopamine D2 receptors in pituitary lactotropes increases body weight and adiposity in female mice. Endocrinology. 2014;155(3):829‐839. 24424036 10.1210/en.2013-1707

[23] Ramos EJ , Meguid MM , Campos AC , Coelho JC . Neuropeptide Y, alpha‐melanocyte‐stimulating hormone, and monoamines in food intake regulation. Nutrition. 2005;21(2):269‐279. 15723758 10.1016/j.nut.2004.06.021

[24] Yoon YR , Baik JH . Melanocortin 4 receptor and dopamine D2 receptor expression in brain areas involved in food intake. Endocrinol Metab (Seoul). 2015;30(4):576‐583. 26790386 10.3803/EnM.2015.30.4.576 PMC4722414

[25] Hagman J , Gralla J , Sigel E , et al. A double‐blind, placebo‐controlled study of risperidone for the treatment of adolescents and young adults with anorexia nervosa: a pilot study. J Am Acad Child Adolesc Psychiatry. 2011;50(9):915‐924. 21871373 10.1016/j.jaac.2011.06.009 PMC3171450

[26] Kafantaris V , Leigh E , Hertz S , et al. A placebo‐controlled pilot study of adjunctive olanzapine for adolescents with anorexia nervosa. J Child Adolesc Psychopharmacol. 2011;21:207‐212. 21663423 10.1089/cap.2010.0139

[27] Lebow J , Sim LA , Erwin PJ , Murad MH . The effect of atypical antipsychotic medications in individuals with anorexia nervosa: a systematic review and meta‐analysis. Int J Eat Disord. 2013;46:332‐339. 23001863 10.1002/eat.22059

[28] Fazeli PK , Calder GL , Miller KK , et al. Psychotropic medication use in anorexia nervosa between 1997 and 2009. Int J Eat Disord. 2012;2012(45):970‐976. 10.1002/eat.22037 PMC3726215 22733643

[29] Herpertz‐Dahlmann B , van Elburg A , Castro‐Fornieles J , Schmidt U . ESCAP expert paper: new developments in the diagnosis and treatment of adolescent anorexia nervosa–a European perspective. Eur Child Adolesc Psychiatry. 2015;24(10):1153‐1167. 26226918 10.1007/s00787-015-0748-7 PMC4592492

[30] Marzola E , Desedime N , Giovannone C , Amianto F , Fassino S , Abbate‐Daga G . Atypical antipsychotics as augmentation therapy in anorexia nervosa. PLoS One. 2015;10(4):e0125569. 25922939 10.1371/journal.pone.0125569 PMC4414549

